# Farmer Perceptions of Pig Aggression Compared to Animal-Based Measures of Fight Outcome

**DOI:** 10.3390/ani9010022

**Published:** 2019-01-10

**Authors:** Rachel S. E. Peden, Irene Camerlink, Laura A. Boyle, Faical Akaichi, Simon P. Turner

**Affiliations:** 1 Animal Behaviour & Welfare, Animal and Veterinary Sciences Research Group, Scotland’s Rural College (SRUC), West Mains Rd., Edinburgh EH9 3JG, UK; Simon.Turner@SRUC.ac.uk; 2 Institute of Animal Welfare Science, University of Veterinary Medicine, Veterinärplatz 1, 1210 Vienna, Austria; Irene.Camerlink@vetmeduni.ac.at; 3 Teagasc, Pig Development Department, Animal & Grassland Research and Innovation Centre, Moorepark, Fermoy Co., Cork P61 C997, Ireland; Laura.Boyle@teagasc.ie; 4 Land Economy Environment and Society Research Group, Scotland’s Rural College (SRUC), West Mains Rd., Edinburgh EH9 3JG, UK; Faical.Akaichi@SRUC.ac.uk

**Keywords:** aggression, animal welfare, desensitization, perception, pigs

## Abstract

**Simple Summary:**

Aggression between pigs is a major animal welfare issue in commercial farming, however only a minority of farmers believe that aggression is a problem that needs to be addressed. We investigated whether the farmers’ reluctance to reduce aggression is linked to desensitization as a result of their frequent exposure to the behavior. We showed farmers video clips of pigs during and immediately after a fight and they judged through a questionnaire the severity of what they saw. These judgments were compared to (a) animal-based measures of injury (skin lesions) and exhaustion (blood lactate), and (b) human observers with and without experience of working with pigs. Farmers perceived fights as severe and were motivated to prevent them continuing. They were not desensitized to aggression as their judgments were similar to those of participants who had never worked with pigs. When farmers (and comparison groups) did not see the fight occurring, they judged exhaustion and injuries to be lower than indicated by the animal-based measures. Farmers could benefit from information on how to better assess the impact of aggression by scoring lesions and from evidence of the economic and welfare impact of these lesions.

**Abstract:**

Several animal welfare issues persist in practice despite extensive research which has been linked to the unwillingness of stakeholders to make changes. For example, most farmers do not perceive pig aggression to be a problem that requires action despite the fact that stress and injuries are common, and that several solutions exist. Frequent exposure to animal suffering could affect farmer responses to distressed animals. This study investigated for the first time whether this occurs, using pig aggression as a focus. Using video clips, 90 pig farmers judged the severity of aggression, level of pig exhaustion and the strength of their own emotional response. Their judgments were compared to objective measures of severity (pigs’ skin lesions and blood lactate), and against control groups with similar pig experience (10 pig veterinarians) and without experience (26 agricultural students; 24 animal science students). Famers did not show desensitization to aggression. However, all groups underestimated the outcome of aggression when they did not see the fight occurring as compared to witnessing a fight in progress. We suggest that farmers be provided with evidence of the economic and welfare impact of aggression as indicated by lesions and that they be advised to score lesions on affected animals.

## 1. Introduction

Farmers are frequently exposed to a range of animal welfare issues, yet they are often unwilling to implement recommendations to improve animal welfare [1,2,3]. It is known that frequently witnessing human suffering can disrupt human emotional, cognitive and behavioral responses to witnessing distress [4,5]. The current study investigates for the first time whether exposure to animal suffering disrupts farmer responses to animal suffering, using pig aggression as a case study. 

Desensitization is a well-established defense mechanism which occurs automatically and unconsciously [4,5]. For example, regular exposure to violence can lead to a reduced emotional response to violence [6], reduced empathy for the victims of violence [4] and increased violent behavior [7]. When witnessing human suffering, the decision to intervene is determined firstly by perceiving there to be an urgent problem that needs to be addressed, followed by feeling personal responsibility to act [8]. Desensitization can interfere with this decision-making process by making incidents less likely to be noticed, by reducing the perceived seriousness of the suffering and by reducing feelings of personal responsibility [6]. It has previously been noted that agricultural communities may become desensitized to animal suffering as they are exposed to it on a regular basis [5]. However, this hypothesis has never been empirically studied despite potentially having important implications for animal welfare and farm efficiency. 

Pig aggression is common in commercial farming as pigs fight to establish dominance relationships following regrouping [9]. In the UK and Ireland, growing pigs are typically regrouped at least once per production cycle, but this can reach as many as four times [10,11], whilst sows are returned to group housing during each gestation [12]. Regrouping occurs to optimize the use of space and to maintain homogeneity in groups (e.g., similar body weight or same gestational phase). Therefore, most farmers regroup animals regularly, and the exact frequency depends on the management of pig batches and farm size. As a result, intensive pig farmers will frequently witness animal suffering due to aggression during their working lives. Aggression between pigs often results in stress for the animals, which can compromise their growth performance [13,14,15], reproductive success [16,17,18] and immune competence [19,20], whilst injuries can impact upon carcass quality [21,22]. However, a recent survey of 167 UK pig farmers revealed that the majority of farmers did not perceive aggression between unfamiliar pigs to be a problem that needs to be addressed [10]. Furthermore, only a minority of farmers attempt to control aggression when regrouping, despite the existence of several effective aggression mitigation strategies [23,24]. These strategies require farmers to make specific changes to animal management or nutrition. For example, allowing litters to mix prior to weaning, housing pigs in large social groups, and enhancing levels of tryptophan in the feed can all reduce the occurrence or intensity of aggression at regrouping [23,25]. It is, therefore, possible that farmers underestimate the impact that aggression has on the welfare and productivity of their animals. The current study aims to investigate: (1) whether farmers underestimate the physical impact of pig aggression and; (2) if this response is influenced by the amount of experience of working with pigs.

## 2. Materials and Methods

### 2.1. Overview

We asked 90 farmers for their perceptual and emotional response to six video clips of aggressive encounters between pigs, employing a paper-based survey. Control groups of non-farmers with experience of working with pigs (10 pig veterinarians) and without experience of working with pigs (26 agricultural students and 24 animal science students) completed an amended version of the survey. Farmers’ scores were compared against the scores of the comparison groups and against objective measures of severity (relative change in number of skin lesions and blood lactate as a result of the interaction) in order to investigate whether farmers underestimate the physical impact of aggression on pig welfare, and how the amount of experience of working with pigs may influence perceptions. 

### 2.2. Ethical Approval

All animal experimentation was approved by Scotland’s Rural College’s (SRUCs) Animal Ethics Committee and the U.K. Government Home Office, ensuring compliance with EC Directive 86/609/EEC for animal experiments. This study was conducted in accordance with the Declaration of Helsinki. This study received internal ethical approval from the Human Ethical Review Committee at the University of Edinburgh (Project identification code: HERC_88_17), and informed consent was obtained for all participants. 

### 2.3. Selection of Video Clips

Video footage was obtained from a separate research project carried out in 2015 at Scotland’s Rural College (SRUC) whereby 168 growing pigs were video recorded in dyadic encounters comprising aggressive interactions. For each pig, measures of skin lesions and blood lactate were taken pre- and post-encounter to indicate relative change as a result of aggression. Skin lesions as a result of receiving bites (i.e., bite marks) are a good indicator of the severity of aggression [21]. Skin lesion count is a validated proxy measure for aggression that is moderately heritable and has been applied in animal welfare assessments [21,26]. Blood lactate gives a measure of physical fatigue. Further details of the dyadic encounters, lesion recording and lactate measurements are provided in [27].

A stepwise selection process was adopted to identify six video clips to be shown to observers. First, pigs that displayed a negative relative change in blood lactate, and therefore displayed a reduction in blood lactate following the fight, were eliminated from the dataset (*n* = 26, see Table 1 for descriptive statistics of the remaining dataset). Second, based on the severity of skin lesions and blood lactate, we identified from the remaining dataset the encounters in which both pigs obtained high (upper quartile, UQ), medium (interquartile range, IQR) or low (lower quartile, LQ) severity measures. Video clip 1 displayed a medium severity mutual fight and was always seen first. This ‘dummy’ clip acted as a practice and a common start point. Moreover, by displaying a typical aggressive encounter, this clip sets the scene for the following experimental clips. Clips 2–4 displayed pigs of low, medium and high severity encounters immediately after the fight ended. Videos of pigs with lesions and lactate in the IQR were also selected showing behavior during the actual occurrence of a fight or during bullying (winner chases the loser) to account for the different types of aggression seen on farms (clips 5–6). This ensured that all observers viewed fights that had ended and interactions that were in progress. The severity and content of each 20 s video clip can be seen in Table 2. Participants were asked to focus on one specific pig. The focal pig obtained severity measures which were as similar as possible to those of the non-focal pig. For exact measures of lesion score and blood lactate for both the focal and non-focal pigs, see Table 3. For a detailed description of the stepwise selection process and criteria, see Appendix A.

The order effects across the observation sessions were controlled for by creating six clip orders, as outlined in Table 4. Footage was edited using Windows Movie Maker (version 2012) and each clip was selected to be 20 s long and such that the focal pig was clearly identifiable. The clips were selected towards the end of the aggressive encounter (clips 1, 5 and 6) or immediately after (clips 2, 3 4), such that the behavior performed was as closely matched in time to the measures of lesions and lactate as possible. Images were played back with sound during observer scoring sessions. Before the clip began, a ‘freeze-frame’ showed the focal pig circled alongside a message stating ‘*please focus on this pig*’. Furthermore, during the clip, every time the focal pig made a major change to its position an arrow appeared pointing towards it.

### 2.4. Survey Design

Answer sheets contained two main sections. Section 1 entitled ‘demographics’ collected information on the farmer’s age (year of birth), gender, role on the farm, farm size and years of experience working with pigs. Farmers were also asked: ‘Do you ever intervene during aggressive encounters between pigs on your farm? (Please tick ALL statements that you agree with from: No, there is no point; No, I never see aggressive encounters on my farm; No, it is too dangerous; Yes, when profitability is likely to be affected, and; Yes, to reduce injuries/stress for the animals)’. Section 2, entitled ‘videos’, was completed alongside watching the assigned movie. Following each video clip, the movie was paused and farmers were asked to place a downward line through three separate 100 mm visual analogue scales (VAS) at a point they felt best represented: (i) how much of a negative emotional reaction they had, from no negative reaction to strongest possible negative reaction; (ii) how exhausting they believed the fight was for the focal pig, from not exhausting at all to the most exhausting possible; (iii) how severe they believed the fight was for the focal pig, from not severe at all to the most severe seen on farms. Additionally, participants were asked what factors they used to judge the severity of the fight (‘Tick all relevant factors from: number/severity of skin lesions, vocalizations, panting, other sounds (e.g., banging), facial expression, stress and others’). For clips 1, 5 and 6 (during fights), farmers were also asked; (iv) if they saw this fight on their farm, how much they would want to prevent it continuing, from not at all to the most possible. Section 1 and Section 2 were amended slightly for non-farmers by removing all farm-related questions and replacing them with those relevant to the control group. For example, questions regarding their role on the farm and farm size were excluded and participants were alternatively asked about their occupation. Participants were instructed not to talk to each other in order to avoid possible effects of their discussions on their answers. For farmer and non-farmer response sheets, see Supplementary Materials.

### 2.5. Recruitment

Participants were recruited between February 2017 and November 2017. Ninety pig farmers were recruited whilst participating in six discussion group events held in the UK and Ireland, organized by Scotland’s Rural College (*n* = 26), Teagasc (*n* = 29) and the Agricultural and Horticultural Development Board (AHDB) Pork (*n* = 35). Ten specialized pig veterinarians participated at the same discussion groups. Veterinarians provide an interesting comparison group due to their comparable years of experience working in the pig industry as the farmers. Farmers and veterinarians were unaware that they would be asked to participate in a study on pig aggression prior to attending the discussion groups. Sixty-one students participated in twelve groups following lectures at SRUC; 35 were students of Agriculture, and 26 studied Animal Science. The student populations provide interesting comparison groups due to their knowledge of farming and livestock, but lack of experience working directly with pigs. Students were unaware that they would be asked to participate in a study on pig aggression prior to attending the lectures. The order of presentation of video clips in Table 4 was replicated twice for students since there were 12 groups compared to the 6 groups of farmers and veterinarians. All responses were collected through ‘face to face’ recruitment; this does not allow response rate calculations. Furthermore, we had no control over the composition of the groups with respect to occupation, so it was not possible to balance each clip order to have the same number of people from each occupation (Table 5).

### 2.6. Demographics of the Final Sample

Nine agricultural students and two animal science students were excluded from the analysis due to reporting prior experience of working with pigs. In total, 150 participants with the following demographics were included:

(1) Pig farmers (*n* = 90) were mostly male (93.3%; female: 6.7%) and were on average 41.5 years old (s.d. = 14.13, range = 17–81 years) with 19.53 years of experience working with pigs (s.d. = 14.74, range = 0.5–65 years). There was a strong, positive correlation between years of experience and age (r = 0.871, *p* < 0.0001). Therefore, only age was included in the statistical analysis but it was considered informative of both age and experience effects. Farmers were mainly farm workers (41.1%), owners (32.2%) and managers (17.8%). The remaining farmers were contract farmers (6.7%) and retired (2.2%). A total of 28.9% were based in Scotland, 38.9% were based in England and 32.2% in Ireland. Additionally, 86.7% of farmers reported currently keeping sows, whilst 67.8% kept weaners (i.e., recently weaned piglets), 55.6% kept growers and 62.6% kept finishers. Therefore, most farmers kept pigs at more than one stage of production. The mean number of pigs kept at each stage of production can be found in Table 6.

(2) Specialized pig veterinarians (*n* = 10) were mostly female (70%; male: 30%) and were on average 40.6 years old (s.d. = 14.4, range = 24–64 years) with 15.3 years of experience working with pigs (s.d. = 17.6, range = 0.75–40 years). Of this group, 40% were based in Scotland and 60% were based in England.

(3) Agricultural students (*n* = 26; 46.2% male, 53.8% female; mean age = 21.4 years, s.d. = 1.65, range = 20–28) were in their 3rd (*n* = 23) and 4th (*n* = 3) years of study.

(4) Animal science students (*n* = 24; 16.7% male, 83.3% female; mean age = 22.2 years, s.d. = 2.97, range = 20–35) were in their 3rd (*n* = 15) and 4th (*n* = 8) years of study. All students were based in Scotland.

### 2.7. Statistical Analysis

Statistical analyses were conducted in Statistical Package for the Social Sciences (SPSS, version 25, International Business Machines Corp., Armonk, NY, USA). Normality of the data was assessed by inspection of the residuals and data were transformed wherever necessary. Residual maximal likelihood (REML) models were run to investigate the factors that influence: (1) Emotional response; (2) Judgment of fight severity; (3) Judgment of exhaustion; and (4) Motivation to intervene if the interaction had occurred on their own farm. The fixed effects in the first three models were gender, age, occupation and the video clip. Occupation was not included as a fixed effect in the fourth model as only farmers were asked the question ‘If you saw this fight on your farm, how much would you want to prevent it continuing?’. The clip order was included in each model as a random effect. The main effects were removed if *p* > 0.1 and the model was re-run until the simplest model was achieved. Post hoc analyses were conducted using least significant difference (LSD) tests with a Bonferroni correction made for multiple comparisons. Six Chi Square tests were carried out to ascertain the effects of occupation on the cues used to judge fight severity. The dependent variables were the use of: (1) lesions; (2) vocalizations; (3) panting; (4) other sounds (e.g., banging); (5) facial expression and; (6) stress when judging fight severity. The results were considered statistically significant where *p* < 0.05.

## 3. Results

A total of 78.9% of farmers indicated that they do intervene when they see pigs fighting on their own farm; 13.3% indicated that they did so when profitability was likely to be affected and 76.7% did so to avoid injuries/stress for the animals. Furthermore, 15.6% of farmers reported that they did not intervene during aggressive encounters on their farm; 7.8% believed there was no point, 2.2% never see aggressive encounters on their farm and 5.6% believed it is too dangerous.

The clip order had no effect on emotional response, judgment of severity, judgment of exhaustion or motivation to intervene. The results of all the main effects are described below.

### 3.1. Main Effects of Occupation

**Figure 1:**
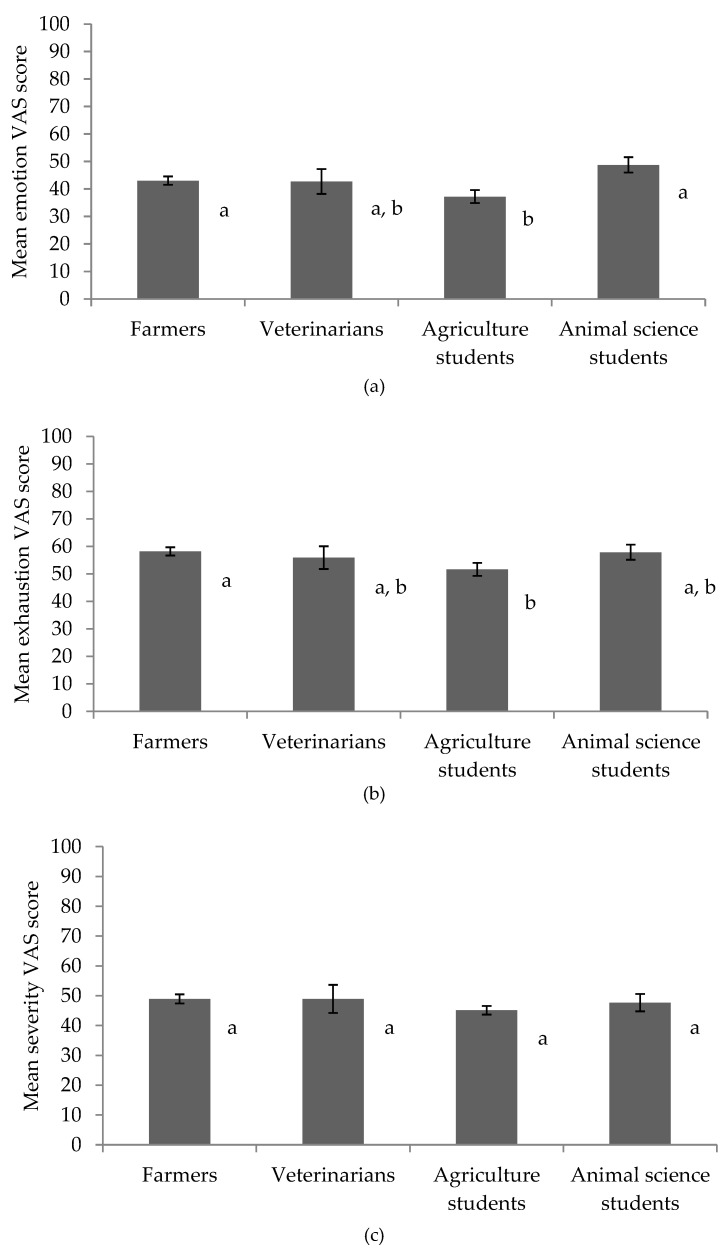
Mean visual analogue scale (VAS) scores for (**a**) emotion, (**b**) exhaustion, and (**c**) severity according to occupation; whereby occupations with different letters express a significant difference in mean response.

**Figure 2:**
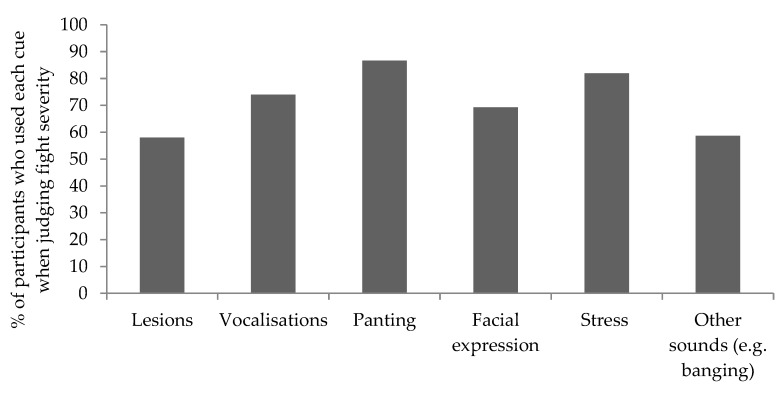
Percentage of participants who used each cue when judging fight severity.

There were significant main effects of occupation on emotional response and judgment of exhaustion across video clips (Table 7). Pairwise comparisons revealed that farmers and animal science students expressed greater emotional response scores when compared to agricultural students (*p* < 0.05; Figure 1a). Farmers judged fight exhaustion to be higher than agricultural students (*p* < 0.01; Figure 1b). Occupations did not differ in their judgments of severity (*p* > 0.05) (Figure 1c). Participants employed a range of cues when judging severity (see Figure 2). There was no effect of occupation on use of skin lesions, vocalizations, panting, facial expressions or stress when judging the severity of aggressive encounters (*p* > 0.05). However, there was an effect of occupation on use of ‘other sounds (e.g., banging)’ (*p* < 0.01), with animal science students using this cue significantly more than farmers and agricultural students (*p* < 0.05).

### 3.2. Main Effects of Video Clip

**Figure 3:**
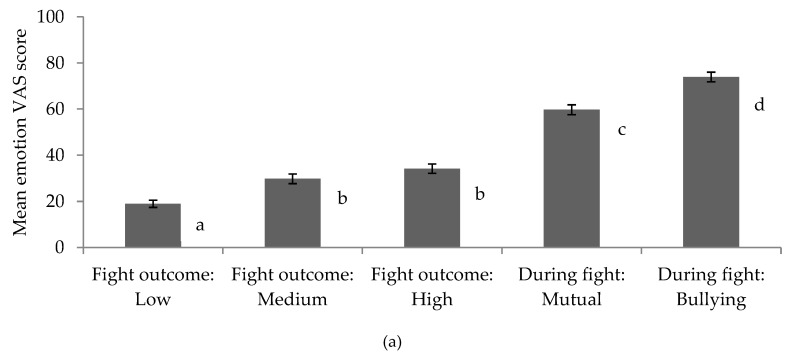

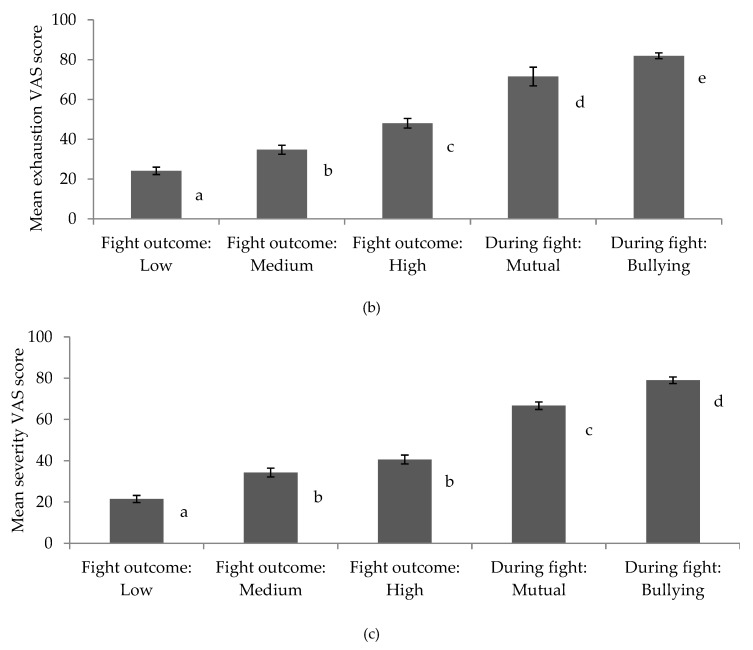
Mean visual analogue scale (VAS) score for (**a**) emotion, (**b**) exhaustion, and (**c**) severity according to video clip; whereby video clips with different letters express a significant difference in mean response.

There were significant main effects of video clip on emotional response, exhaustion score, severity score and farmer motivation to intervene across occupations (Table 7). The mean emotional response, exhaustion score and severity score for the low severity outcome clip (lower quartile lactate and lesions) showing pigs after a fight (clip 2) were significantly lower than for the medium and high severity outcome clips (clips 3 and 4), as well as for the mutual fight and the bullying clips (clips 5 and 6; *p* < 0.01). Emotional response and severity scores for the medium and high severity outcome clips showing pigs after a fight (clips 3 and 4) did not differ (*p* > 0.05) but the exhaustion scores did (*p* < 0.001), whereby pigs with a higher lactate level and more lesions were regarded as being more exhausted. Emotional response, exhaustion score and severity score for both of the ‘during fight’ clips (clips 5 and 6) were greater than the scores for the ‘post-fight’ clips, and responses to the bullying clip (clip 6) were significantly greater than to the mutual fight clip (clip 5) (*p* < 0.001; Figure 3a–c). Farmer motivation to intervene was significantly greater for the bullying clip than for the mutual fighting clip (Bullying: mean = 79.4, SE = 2.4; Mutual fight: mean = 72.0, SE = 2.5; *p* < 0.01).

### 3.3. Demographic Effects

Across occupations, women expressed significantly greater VAS scores compared to men for emotional response (Females: mean = 46.1, SE = 2.0; Males: mean = 41.6, SE = 1.4; *p* < 0.01), judgment of exhaustion (Females: mean = 59.2, SE = 1.9; Males: mean = 55.9, SE = 1.4; *p* < 0.01), and judgment of severity (Females: mean = 50.0, SE = 2.0; Males: mean = 47.2, SE = 1.4; *p* < 0.05). Female farmers also expressed greater motivation to intervene than male farmers (Females: mean = 88.8, SE = 3.5; Males: mean = 74.8, SE = 1.8; *p* < 0.05). There was a significant effect of age on farmer motivation to intervene (*p* < 0.05) with older farmers expressing greater motivation to intervene, although the significant positive correlation was weak (r = 0.148, *p* < 0.05). There was a significant effect of age on judgment of severity (*p* < 0.01) most likely linked to a cohort of young participants (agriculture students) who gave lower scores.

## 4. Discussion

It is known that frequent exposure to suffering can disrupt human emotional, cognitive and behavioral responses to signs of distress [4,5], and the current study provides the first investigation into whether or not routine exposure to animal suffering disrupts farmer responses, using pig aggression as a case study. Our survey amongst 90 farmers and 60 control participants showed that farmers are not desensitized and in fact are motivated to change the situation when noticed. All participants assessed aggression as severe when having seen the fight, but underestimated the impact of aggression when the animals were viewed immediately after the fight had ended (assessed through objective animal-based measures).

### 4.1. Perceptions of Aggression

All comparison groups judged aggressive behavior in-action to be highly severe and exhausting for the animals, and experienced a negative emotional response to these interactions. Responses were particularly high for bullying aggression in comparison to mutual aggression, despite both encounters resulting in medium severity measures of blood lactate and skin lesions. Farmers reported that, if they saw these interactions on their farm, they would be highly motivated to prevent them continuing. Indeed, the majority of farmers reported that they do intervene when they see aggressive interactions on their farm, and their primary motivation for doing so was to reduce injuries and stress for the animals. Nevertheless, all comparison groups underestimated the impact of aggression as indicated by skin lesions and blood lactate when they did not see the fight occurring. Specifically, the perceived seriousness of injuries and exhaustion as a result of aggression, as well as judgments of their own emotional response, were lower when observing the animals immediately after a fight had ended, even when the outcomes were severe as indicated by objective measures. Furthermore, judgments of severity and emotional response did not differ between the medium and high severity post-fight outcome clips, suggesting that the participants perceived little difference between these outcomes.

Farmers are often engaged in a wide range of tasks performed in different buildings which makes it difficult for them to witness post-mixing fights as frequently as they actually occur. Farmers are expected to witness the injuries from such interactions during their regular animal inspections. However, results suggest that farmers are unlikely to fully recognize the severity of these outcomes and this may contribute to the limited uptake of recommendations from aggression research in commercial practice.

Farmers were not desensitized to aggression as a result of frequent exposure to the behavior; farmer perceptions of fight severity were comparable to those of participants with, and without, experience of working with pigs, and all participants employed a range of pig-based cues (lesions, facial expression, panting, stress and vocalizations) to a similar extent when making these judgments. Farmers and agriculture students did use ‘other sounds (e.g., banging)’ less than applied animal science students, which suggests that they relied on a more limited set of cues when making their judgments. However, it is unclear why this occurred. Farmers judged exhaustion to be significantly higher than agricultural students. Furthermore, farmers and applied animal science students experienced a greater negative emotional response to aggression when compared to agricultural students. Stakeholders differ in their knowledge, interests, values and norms regarding livestock. This can influence their perceptions of animal welfare [28,29,30] and may have contributed to the lower responses detected for agricultural students.

There were important differences between our comparison groups in their age and gender, and results confirmed that it was crucial to control for these differences in statistical analysis. Women on average gave higher scores than men for emotional response, judgment of severity and judgment of exhaustion, and female farmers were more motivated to intervene during fights than male farmers. This is consistent with evidence that, on average, females show more positive behaviors and attitudes toward animals; for example, by expressing greater empathy for animals [31,32], more opposition to animal use, and greater involvement with animal protection activities [33,34]. Furthermore, older farmers expressed greater motivation to intervene than younger farmers. Therefore, as age and years of experience were highly related, farmer experience may actually enhance their responses to fights. Age also influenced participant judgments of severity, which was determined by a subgroup of young participants who gave lower scores (agriculture students).

### 4.2. Animal Welfare Implications

The results indicate two important targets for implementing a change in practice. Firstly, farmers must be made aware of how to accurately determine the physical impact of aggression when they have not witnessed the fighting behavior. One useful tool for farmers to achieve this is scoring or estimating the number of visible lesions on affected animals. Counting lesions, or the simplified skin lesion score method, is an established and accurate measure of aggressive behavior which is regularly employed in research [21,35]. Secondly, researchers should calculate the economic and welfare impact of aggression as indicated by the lesions. If farmers observe the true frequency and intensity of fighting behavior on their own farm, and understand its impact on farm productivity, their motivation to control the issue is likely to increase. This advice regarding the recognition of aggression as a problem should be translated effectively to farmers and other stakeholders within the industry. Veterinarians are particularly important as they are the most valued source of information to farmers and highly influential in determining their animal welfare decisions [11,36].

### 4.3. Evaluation of Novel Methodology

The current study employed a novel methodology whereby perceptions of aggression exhibited in video clips were compared to objective, physiological measures of the severity of the welfare threat. There are many other animal welfare issues that have been resistant to change despite extensive research [3,37,38]. This novel methodology may represent a useful tool to assist in establishing stakeholder perceptions of these issues, in order to tailor successful interventions. There are a number of evaluative points regarding this technique, which are important to highlight here. First, ecological validity is limited by the use of video clips, which are removed from the real farm setting. However, the use of video footage with corresponding data allowed careful control over the experimental stimulus, which would not be possible in a real farm setting. Furthermore, by using pre-existing footage and data, we were able to avoid the use of animals for the purpose of the study (supporting the 3Rs of animal research: [39]), and maximize the utilization and impact of existing data. Second, although the self-reported measure of emotional response allowed for quick and easy data collection, this could be influenced by experimenter effects whereby participants might have responded in the way that they thought was being sought rather than how they really felt [40]. Future research could build upon the findings of this study by employing physiological measures of emotional response such as participant heart rate and galvanic skin response, which are less open to bias [41]. Third, the methodology allowed efficient data collection at pre-existing farmer discussion groups, as the procedure could be completed quickly with all group members participating simultaneously. Fourth, the method has quantified how well subjective scoring by observers compares to objective measures of the outcome of aggression (skin lesions and blood lactate). This makes the assumption that the objective measures are a closer approximation to the true experiences of the animal than the subjective scores but it is acknowledged that qualitative scores based on animal demeanor can also reflect welfare [42]. Finally, we did not include a non-professional group (e.g., consumers) as this was not within the aims of the current study. This study focused on how farmer exposure to pig aggression may have influenced their perceptions of aggression relative to others with experience of pigs (veterinarians) or with knowledge of agriculture but little experience of the pig industry (students). However, subsequent research examining consumer perceptions would be valuable.

## 5. Conclusions

Farmers were not desensitized to pig aggression. Farmers experienced a negative emotional response to seeing fights between pigs. They judged fights to be severe and exhausting for the animals and they were motivated to prevent them continuing. However, farmers and other observer groups underestimated the physical impact of aggression when they did not see the fight occurring and this may contribute to the limited uptake of methods to reduce aggression in commercial practice. Farmers are unlikely to see fights as frequently as they actually occur, and this likely limits their perception of aggression as a problem on their farm and their motivation to control aggression. In order to bridge the gap between research and practice, researchers must provide farmers with evidence of the economic and welfare impact of aggression as indicated by lesions, and farmers must be encouraged to estimate the impact of fights on their farm by counting lesions on the affected animals.

## Figures and Tables

**Table 1:** Descriptive statistics regarding measures of relative change in lesions (number of lesions per pig) and blood lactate (mmol/L) following the fight, compared to before the fight, for the dataset (*n* = 142).

Measure	Blood Lactate	Lesion Score
Mean	7.76	57.37
Min	0	0
Quartile 1	2.15	12.75
Quartile 2	42.00	5.85
Quartile 3	13.20	77.25
Max	21.20	354

**Table 2:** Severity and content of each 20 s video clip displaying an aggressive encounter between two pigs. (LQ = lower quartile; IQR = interquartile range; UQ = upper quartile).

Clip	Blood Lactate	Lesion Score	Behavior
1 (‘Dummy’)	IQR	IQR	During mutual fight
2 (‘Fight outcome: Low’)	LQ	LQ	After fight
3 (‘Fight outcome: Medium’)	IQR	IQR	After fight
4 (‘Fight outcome: High’)	UQ	UQ	After fight
5 (‘During fight: Mutual’)	IQR	IQR	During mutual fight
6 (‘During fight: Bullying’)	IQR	IQR	During bullying

**Table 3:** Exact measures of relative change in lesion score (number of lesions per pig) and blood lactate (mmol/L) following the fight, compared to before the fight, for the pigs in each video clip.

Clip	Focal Pig		Non-Focal Pig	
	Blood Lactate	Lesion Score	Blood Lactate	Lesion Score
1	3.4	20	5.1	31
2	0.6	2	0.3	0
3	4.3	55	8.8	30
4	20.8	82	16.8	94
5	9.4	56	5.8	24
6	5	45	12	0

**Table 4:** Each of the six clip orders.

Block	Clip Order					
	A	B	C	D	E	F
1	1	1	1	1	1	1
2	3	4	2	3	4	2
2	4	2	3	4	2	3
2	2	3	4	2	3	4
3	5	5	5	6	6	6
3	6	6	6	5	5	5

**Table 5:** Total number of participants who watched each of the six clip orders.

Clip Order	Total N			
	Farmers	Pig Veterinarians	Agricultural Students	Animal Science Students
A	26	4	6	0
B	9	0	1	8
C	7	1	6	2
D	20	0	4	6
E	20	3	1	2
F	8	2	8	6
Total	90	10	26	24

**Table 6:** Mean number of pigs kept at each stage of production at any one time (in brackets are the number of farmers that kept pigs at the specified stage of production), range and standard deviation (s.d.).

	Mean (Number)	Range	s.d.
Weaners	1929 (61)	150–10,000	1829.12
Growers	2850 (50)	10–30,000	5787.05
Finishers	3835 (56)	100–38,000	7109.85
Sows	1100 (78)	40–13,500	2127.45

**Table 7:** The results of four residual maximal likelihood (REML) models investigating the factors that influence: (1) emotional response; (2) judgment of exhaustion; (3) judgment of severity and; (4) motivation to intervene. The main effects were removed from the model if *p* > 0.1 unless involved in a significant interaction.

Main Effect	F (df)	*p*
**Emotional response**		
Gender	7.0 (1)	0.009
Occupation	4.5 (3)	0.004
Video clip	136.4 (4)	0.001
**Judgment of exhaustion**		
Gender	8.4 (1)	0.004
Occupation	4.8 (3)	0.002
Video clip	131.6 (4)	0.001
**Judgment of severity**		
Gender	6.8 (1)	0.010
Age	8.1 (1)	0.005
Video clip	153.6 (4)	0.001
**Farmer motivation to intervene**		
Gender	4.9 (1)	0.029
Age	4.9 (1)	0.030
Video clip	8.7 (1)	0.004

## Supplementary Materials

Data for this project and the surveys are available at: https://osf.io/rh2sz/?view_only=d8b4c6f08e1448d487a4d204cf43077a.

## Appendix A

**Stepwise selection of video clips**

The final six video clips were selected using the following stepwise selection process:

(1) Video clips were collected based on data obtained from research carried out by an SRUC project in 2015. The descriptive statistics for the full dataset can be found in Table A1.
  **Table A1:** Descriptive statistics regarding measures of relative change in skin lesions (number of lesions per pig) and blood lactate (mmol/L) following the fight, compared to before the fight, for the entire dataset (*n* = 168).
  Measure	Lesion Score	Blood Lactate
  Mean	49.89	6.43
  Min	0	−2.80
  Quartile 1	6.00	0.53
  Quartile 2	30.00	4.20
  Quartile 3	74.75	10.80
  Max	354	21.20
(2) Pigs that displayed a negative relative change in blood lactate, and therefore displayed a reduction in blood lactate following the fight, were eliminated from analysis. In doing this, 26 pigs were eliminated. Descriptive statistics for the remaining dataset can be found in Table 1 (see Methods).
(3) Criteria for the video clips were set based on quartiles. Video criteria can be seen in Table A2.
  **Table A2:** Description of criteria used to identify video clips (LQ = lower quartile; IQR = interquartile range; UQ = upper quartile).
  Clip	Focal Pig		Non-Focal Pig	
  	Lesion Score	Blood Lactate	Lesion Score	Blood Lactate
  1	IQR	IQR	IQR	IQR
  2	LQ	LQ	LQ	LQ
  3	IQR	IQR	IQR	IQR
  4	UQ	UQ	UQ	UQ
  5	IQR	IQR	IQR	IQR
  6	IQR	IQR	No criteria set	
(4) Contests that met the criteria for each clip were identified using the ‘select cases’ function in SPSS. The number of video clips to meet the criteria for each video clip can be seen in Table A3.
  **Table A3:** Number of contests to meet the criteria for each video clip.
  Clip	Number of Videos Identified
  1	9
  2	2
  3	9
  4	5
  5	9
  6	34
(5) All potential video clips were then watched to identify the six clips that best matched the selection criteria described in Table A4.
(6) Video clips were selected and Table 3 provides the exact objective measures for each of the pigs observed in the six video clips (see Methods).
  **Table A4:** Description of video clip selection criteria and content (LS = lesion score).
  Clip	Behaviour	Selection Criteria
  1	*During fight:*	Both pigs engaged in mutual fighting behavior for a minimum period of 20 s.
  		Both pigs remained in view of the camera for the duration of the 20 s.
  		Both pigs obtained medium severity LS and lactate measures following the fight.
  2	*After fight:*	During the 60 s immediately *after the fight ended*, both pigs were in view for a minimum period of 20 s.
  		Both pigs displayed low severity LS and lactate measures following the fight.
  3	*After fight:*	During the 60 s immediately *after* the fight, both pigs were in view for a minimum period of 20 s.
  		Both pigs displayed medium severity LS and lactate measures following the fight.
  4	*After fight:*	During the 60 s immediately *after the fight ended*, both pigs were in view for a minimum period of 20 s.
  		Both pigs displayed high severity LS and lactate measures following the fight.
  5	*During fight:*	During the 60 s immediately *before the fight ended*, both pigs engaged in mutual fighting behavior for a minimum period of 20 s.
  		Both pigs remained in view of the camera for the duration of the 20 s.
  		Both pigs obtained medium severity LS and lactate measures following the fight.
  6	*During fight:*	During the 60 s immediately *before the fight ended*, one pig displayed bullying behavior whilst the other attempted to retreat for a minimum period of 20 s.
  		Both pigs remained in view of the camera for the duration of the 20 s.
  		The focal pig (recipient of bullying) obtained medium severity LS and lactate measures following the fight.
  		No criteria were set for the non-focal (bullying pig) with regards to LS and lactate measures. This was because the two very different behaviors cannot be expected to result in the same measures.
